# Conflicts between healthcare professionals and families of a multi-ethnic patient population during critical care: an ethnographic study

**DOI:** 10.1186/s13054-015-1158-4

**Published:** 2015-12-22

**Authors:** Rose-Lima Van Keer, Reginald Deschepper, Anneke L. Francke, Luc Huyghens, Johan Bilsen

**Affiliations:** Mental Health and Wellbeing Research Group (MENT), Department of Public Health, Faculty of Medicine and Pharmacy, Vrije Universiteit Brussel, Laarbeeklaan 103, 1090 Brussels, Belgium; NIVEL, Netherlands Institute for Health Services Research, EMGO+/VU University Medical Center, Postbus 1568, 3500 BN Utrecht, The Netherlands; Critical Care Department/Service of Intensive Care Medicine, Vrije Universiteit Brussel, Universitair Ziekenhuis Brussel, Laarbeeklaan 101, 1090 Brussels, Belgium

**Keywords:** Intensive care, Cultural diversity, Ethnic minorities, Communication, End-of-life decision making, Communication barriers, Conflict

## Abstract

**Background:**

Conflicts during communication in multi-ethnic healthcare settings is an increasing point of concern as a result of societies’ increased ethno-cultural diversity. We can expect that conflicts are even more likely to arise in situations where difficult medical decisions have to be made, such as critical medical situations in hospital. However, in-depth research on this topic is rather scarce. During critical care patients are often unable to communicate. We have therefore investigated factors contributing to conflicts between healthcare professionals and family members from ethnic minority groups in critical medical situations in hospital.

**Methods:**

Ethnographic fieldwork was done in one intensive care unit of a multi-ethnic urban hospital in Belgium over 6 months (January 2014 to June 2014). Data were collected through negotiated interactive observation, in-depth interviews with healthcare professionals, from patients’ medical records, and by making notes in a logbook. Data were analysed by using grounded theory procedures.

**Results:**

Conflicts were essentially related to differences in participants’ views on what constitutes ‘good care’ based on different care approaches. Healthcare professionals’ views on good care were based predominantly on a biomedical care model, whereas families’ views on good care were mainly inspired by a holistic lifeworld-oriented approach. Giving good care, from the healthcare professionals’ point of view, included great attention to regulations, structured communication, and central decision making. On the other hand, good care from the families’ point of view included seeking exhaustive information, and participating in end-of-life decision making. Healthcare professionals’ biomedical views on offering good care were strengthened by the features of the critical care context whereas families’ holistic views on offering good care were reinforced by the specific characteristics of families’ ethno-familial care context, including their different ethno-cultural backgrounds. However, ethno-cultural differences between participants only contributed to conflicts in confrontation with a triggering critical care context.

**Conclusions:**

Conflicts cannot be exclusively linked to ethno-cultural differences as structural, functional characteristics of critical care substantially contribute to the development of conflicts. Therefore, effective conflict prevention should not only focus on ethno-cultural differentness but should also take the structural organizational characteristics of the critical care context sufficiently into account.

**Electronic supplementary material:**

The online version of this article (doi:10.1186/s13054-015-1158-4) contains supplementary material, which is available to authorized users.

## Introduction

Critical medical situations, i.e. situations involving far-reaching decisions about the life and death of a seriously ill patient, are very challenging for healthcare professionals. Moreover, discussing such crucial decisions with critically ill patients often becomes impossible due to their incompetence, resulting in an increased need for optimal communication about these issues with the relatives or legal representatives of the patient [1–3]. Confidence and trust are seriously challenged, and conflict can easily arise [4, 5]. A recent study suggests that conflicts between physicians and patients’ substitute decision makers occurs in nearly two-thirds of cases [6].

Conflicts between healthcare professionals and relatives regularly occur in matters such as whether or not to limit life-sustaining treatment, the appropriate time for patients to be discharged from the intensive care unit (ICU) and the patients’ death [5–9]. Communication between relatives and healthcare professionals can become seriously challenged due to the predominantly technological orientation in critical care settings, combined with the uncertainty, anxiety and moral dilemmas, experienced in such situations [1–3, 7]. Families’ inability to fully understand patients’ prognoses, their unrealistic expectations towards staff resulting from media exposure as well as a late and incomplete integration of patients’ substitute decision maker into end-of-life discussion can further hinder effective communication with healthcare professionals. Also, physicians’ lack of communication skills, their uncertainty about their own clinical judgement, job strain as well as intra-team conflicts can impede adequate communication with families [4, 7, 8, 10]. Such conflicts may considerably jeopardize the provision of adequate care for the patient, but can also endanger the critical care team’s wellbeing and cohesion [11]. Therefore it is important to further investigate the types of conflict faced by healthcare professionals during their contact with family members [11–13].

Healthcare professionals increasingly often have to communicate with family members from ethnic minority groups, i.e. persons of a different origin who share certain cultural characteristics to some extent [14], as a result of societies’ increased ethno-cultural diversity. It is recognized that families from ethnic minority groups are at a higher risk of stress and potential conflict than families from the ethnic majority group due to their different ethno-cultural background [15–18]. Communication challenges and conflicts can occur with relatives from ethnic minority groups around critical medical decision making, communication of bad news and the more practical aspects of caring for the patient. Incongruent beliefs about the causes and treatment of an illness, language difficulties, strong religious beliefs and ethno-cultural norms and values (e.g. gender values) are often described as root causes of tensions. Additionally, healthcare professionals’ lack of knowledge about ethno-cultural differences and ethnic stereotyping can further hinder trustful communication with relatives [15, 16, 19–23]. However, although we live in an increasingly multi-ethnic society, in-depth research about the extent to which ethno-cultural differences (e.g. linguistic, religious differences) contribute to these conflicts during daily encounters between healthcare professionals and families is rather scarce. Policy makers state that there is a pressing need to develop ‘migrant-friendly hospitals’ in Europe, as declared in the Amsterdam Declaration [24].

In this study, we therefore aim to investigate ‘the factors contributing to conflicts between healthcare professionals and families from ethnic minority groups in a multi-ethnic ICU’. Understanding conflicts between healthcare professionals and family members of critically ill patients from ethnic minority groups can serve as a first step in the development of recommendations for preventing and resolving conflict.

## Methods

‘Conflict in a multi-ethnic critical medical care setting’ is a sensitive, complex and novel topic of research. Therefore an ethnographic research design was used, consisting of negotiated interactive observation [25, 26], in-depth interviews [27], the reading of patients’ medical records, and making notes in a logbook [27, 28].

### Participants and setting

Ethnographic fieldwork was done in one ICU of a multi-ethnic urban hospital in Belgium over 6 months (January 2014 to June 2014). Staff and relatives’ behaviour and interaction in the ICU was studied for 360 hours by the principal researcher. The selected patients and the family members who accompanied them, as well as their healthcare professionals, were followed for the whole critical period, namely from patients’ admission until death or discharge from the ICU. Patients and their family members were purposefully selected. They were only eligible for inclusion in the study if the patient or at least one of his/her legal parents was born abroad, if at least one of the family members was able to speak Dutch, French, or English, and if the patient was at least 18 years old. In total we selected 10 patients and their visiting family members, who were originally from North Africa, Turkey, Central Africa and Southern Europe, which are the regions of origin of large ethnic minority groups in Belgium [29]. The patients were between 40 and 82 years old and consisted of six males and four females. They were dependent on intensive care for between 1 and 15 weeks.

The critical care team consisted of 80 nurses and 12 doctors, who were almost all white Caucasians from the dominant ethnic group. A total of 39 % were men, 61 % were women. A total of 42 % were under 45 years old and 10 % had their place of residence in the urban area where the hospital was located.

### Data collection

Data were collected through triangulation of several data collection strategies, namely negotiated interactive observation on the ward [25, 26], in-depth interviews [27], the reading of patients’ medical records, and the writing up of the researcher’s reflections on her behaviour and feelings in the field in an ethnographic logbook [27].

In an ICU setting, typified, among other things, by anxiety and highly specialized and delicate work, it is very difficult for the researcher to fully participate in the core activities of social life on the ward. Therefore ‘negotiated interactive observation’ was chosen, meaning that the researchers’ observations went hand in hand with an implicit or explicit negotiation of them with the research participants [25, 26]. For example, before attending a bad news conversation between relatives and doctors, the researcher always asked both parties for permission to attend the conversation. This technique enabled the researcher to gain the trust of research participants easily. Consequently, negotiated interactive observation gave the researcher the opportunity to have 432 informal conversations with healthcare professionals and family members, attend 144 staff meetings and witness 288 interactions between healthcare professionals and relatives during visiting hours.

When the researcher was in the ICU she made notes, i.e. key words or short phrases, in a logbook during or right after her observations and communication with the research participants. After the researcher had left the ICU, she transformed these notes into comprehensive, descriptive field notes containing both observations of participants’ discourse and behaviour in and beyond situations of conflict and informal conversations with the participants. Registering participants’ discourse and behaviour in conflict situations is considered more relevant than what participants told the researcher they would say and do during conflicts. Formal in-depth interviews were held with nine healthcare professionals (Topic list for formal in-depth interviews: see Appendix). These interviews were recorded. In order not to increase the burden on relatives, no formal in-depth interviews were held with them. All data were collected by the first author (RVK), a trained ethnographer and sociologist.

### Data analysis

The analysis started with a ‘thick description’ [30] of the social interactions on the ward and was followed by a grounded theory analysis [31, 32]. In-depth interviews were transcribed, and data were conceptualized via a three-step coding process (open coding, axial coding, and selective coding), supported by the qualitative data analysis software package NVIVO 8 (QSR International, 2008). This process resulted in the creation of a conceptual model, consisting of different themes and subthemes. First, an open coding phase was performed, involving the reading and rereading of the data. This resulted in the formation of different codes, for instance ‘supportive activities’, ‘comfort-increasing activities’, and ‘safeguarding the clinical state of patient’. To find similarities and differences between these codes, new codes were constantly compared with existing codes. Second, axial coding took place. This led to the formation of groupings of similar codes, i.e. categories, for example ‘bedside care activities’, ‘technical medical care’. Relationships between the categories were also established, for instance ‘holistic care’ and ‘biomedical care’. Third, selective coding was performed to determine the core category (‘views on good care’) around which the related categories are clustered [31, 33].

As usual in qualitative research, the stages of data collection and data analysis were interwoven, and interim analyses were used to facilitate more focused observation and data collection [27, 31–33]. Data collection and analysis were stopped when the point of saturation was reached, i.e. when additional data did not give us any new insights into the topic of conflict [31, 34]. Analysis of atypical cases was important to gain a more comprehensive understanding of the circumstances under which conflict was likely to arise. Reliability was strengthened by the first author (RVK) doing the data analysis and two co-authors (RD and JB) doing a peer revision of the analysis. The process of data collection and data analysis was also regularly discussed by members of the multi-disciplinary academic research group in which these authors participate, consisting of a health scientist, a nurse, two anthropologists and a sociologist. To improve the reliability and accuracy of the study, the results were also read by an intensive care specialist who is part of the intensive care team at the hospital in which the researcher did her fieldwork.

### Ethics

The research protocol (reference 2013/371) was q approved by the university ethics committee of the Vrije Universiteit Brussel. The privacy of the research participants and confidentiality of the data were respected, e.g. by the use of pseudonyms. Written consent to participation in the study was sought from the healthcare professionals, family members, and patients who were still able to communicate. If the patient lacked the capacity to give consent, consent was sought from his or her legal representative.

## Results

Patients were admitted for complicated pneumonia (three), abdominal problems (one), heart problems (two), brain haemorrhage (two), cancer (one), and a severe accident (one). All the patients were sedated for some time, and as a result were unable to communicate or only had limited ability to communicate, depending on the level of sedation.

In nine of the ten cases, one or more conflicts occurred during the period of ethnographic fieldwork. We found that these conflicts were related to families’ and healthcare professionals’ different expectations of (1) care practices, (2) emotional involvement, (3) information exchange and (4) end-of-life decision making (see Additional file 1). In the case where no conflict occurred, the patient received less frequent and fewer visitors than in the other cases.

### Care practices

Families’ care practices, associated with family structures, their shared culture and integration-related role expectations, hindered healthcare professionals in their task of safeguarding patients’ clinical state and controlling the ICU environment and their workload, which contributed to conflict.

#### Visits

Visits to the patient were seen by most visitors as a social or religious duty. As most migrant families were large, patients received many visitors during visiting hours. However, not only relatives but also friends and neighbours came to visit the patient because they considered them family.

A visitor coming to see Norah tells me that he is the son of a friend of the patient’s husband. I ask if he comes to visit the patient daily. He says: “*Yes, I have to because she’s like family.*” [Field note, Norah]

To safeguard patients’ clinical state and respect patients’ and other families’ privacy, healthcare professionals tried to ensure that rules concerning visits were followed. According to these rules, visitors were allowed to come to the patient’s bedside in twos and could not stay with the patient for longer than 1 hour.

Then a man comes into the patient’s quarantine area. The nurses notice this and wonder who the man is, since they have never seen him before. He says he is a friend. A nurse says to the man, “*Only family can come into Intensive Care, not friends.*” [Field note, Zacharia]

“*Come on, I mean, the more distant they were from them, the more, the more they, erm, they, they start causing trouble eh… (…) I’m committed to never getting mixed up in all that business, come on, I mean you’ve got a sick patient, okay, you have family, and you try to look after that patient, and to give the right family members information (…). Sometimes they come with nephews and nieces along with the son, the wife, and then I think, ‘Well if that’s all right with you, it’s all right with me! As long as you don’t turn the place upside down, and only two or three of you are standing around the bed at once!*’” [Interview with nurse Jeannine]

However, the rules were often broken. Some visitors tried to negotiate with healthcare professionals for a flexible application of the rules. Breaking the rules concerning visits made the encounters between visitors and staff members stressful. Some healthcare professionals did not feel at ease when doing care tasks in the presence of visitors or when the latter tried to negotiate with them about these rules.

Nurse: “*Here you have to tell them and keep on telling them. Because often they keep doing it anyway. I wonder how many of them there are here this time.*” She looks over towards the bed where Norah is lying. She stands up and goes for a look. She approaches the family with her hands on her hips. She looks at Norah’s family members who are standing around the bed. The patient’s two daughters and brother are there, so three people instead of the maximum two allowed. The nurse says loudly, “*How often do I have to tell you that only two people are allowed to visit the patient… there are three of you*!” The family members look horrified. The patient’s brother gets up to leave. A daughter looks a bit angry and clearly feels attacked. She says, “*But I have seen that there are three people sitting at other beds*.” Céline sighs, leaves the bed and goes angrily to check the other beds. She comes back to the daughter and says, still in a loud voice, “*I can’t see three people anywhere else… Come and look*!” Céline walks away angrily, and the daughter still looks angry. Céline says, “*You just don’t get it, do you.*” [Field note, Norah]

#### Bedside care activities

Another important activity practiced by families is bedside care activities, which encompassed supportive and comfort-increasing activities. These activities were to a great extent ethno-culturally determined. Families’ supportive activities included affective deeds, for instance touching and kissing the patient, and religious acts. Comfort-increasing activities included giving a massage, bringing along food and refreshing the patient. Visitors’ bedside activities served as a form of communication between them and the patient as they wanted to tell the patient that they were still there for him/her.

Families performed bedside care activities spontaneously or at the patient’s request. Some patients requested care from families because they lacked knowledge of the language of the host society and thus were not able to communicate with healthcare professionals effectively. Patients also requested or consented to family members’ care acts through non-verbal communication, which was understood and reacted to better by families than by healthcare professionals as the latter were occupied with performing urgent lifesaving care tasks and thus experienced time pressure.

I go to Norah’s bed when one of her daughters is also at her mother’s bedside. The breathing tube is still in Norah’s mouth. Norah seems to be asleep but she does react to her daughter’s questions by nodding or opening her eyes slightly. She clearly realises her daughter is nearby. (…) She (the daughter) asks her mother if she should massage her feet. Norah nods. It is strange to see that the patient is ‘asleep’ but still communicating with her daughter. (…) The daughter starts massaging her mother’s feet. (…) After massaging her mother’s feet, she goes on talking to her for a while in Berber. [Field note, Norah]

**Nurse**: “*I felt he, erm, he had times when he, well, called a lot for silly, well, silly things. Particularly if he is in isolation at that point then it’s sometimes, erm, well, a lot of work to go in there, for something silly. You know. Taking your clothes off and washing again. Sometimes I lost a lot of time doing that*.”

**Researcher**: “*Can you give an example of one of the silly things you had to go to him for?*”

**Nurse:** “*Oh, erm, his, a bottle he didn’t for example, well, his urine bottle or something that was five centimetres too far to the right, he wanted it closer.*” [Interview with nurse Vanessa]

Disapproval of these care practices was common among healthcare professionals because they felt these acts sometimes endangered the clinical state of the patient, which led to frustration among some family members.

A little later a young man comes to visit the patient and tries to touch his face. A nurse sees this and tells him not to do it because it is dangerous. The man doesn’t seem to hear her. The woman repeats the information. The man gets angry and says: “*But he’s my uncle!*” (…) The man gets even angrier, and the nurse asks him to leave the ward since he cannot control his temper. [Field note, Abdallah]

#### Care-related requests

A final care practice by families was making care-related demands of healthcare professionals. Visitors requested care from healthcare professionals when they thought or heard from the patient that some of his/her needs were not being met. Because some patients could only express themselves in their mother tongue, or expressed themselves better in that language, they requested care in their mother tongue from their visitors who spoke the same language. Visitors took on the role of translator or assistant who let healthcare professionals know about patients’ wishes. Families’ care-related requests often evoked overt frustration among staff as these demands, often not so urgent, increased their workload. These conflicts diminished visitors’ trust in some healthcare professionals because they were considered aggressive and insufficiently understanding of the patient’s needs.

The daughter looks for another pillow to place under her (the patient’s) other foot, but she cannot find one. Then she asks me if I could ask the nurses to bring a pillow. I go out of quarantine and go and ask the nurses if there is a pillow anywhere. One nurse answers with irritation: “*We have only just laid her down and she wants to sit up again… Now she wants a pillow… This time she will just have to wait. We’ll come in a few minutes… Tell her that…* ” I go back to the daughter and say a nurse will bring a pillow in a few minutes. She says: “*Ah, okay.*” Ten minutes later, still no nurse has come, and the daughter is using a towel as a pillow. She puts the towel under the patient’s feet. The nurse walks past and sees the daughter putting a towel under her mother’s foot and says angrily: “*You’re not allowed to do that… We use special pillows for that… I said I’d come!*” Shocked, the daughter replies: “*Ah*, *I didn’t know, ok…*” She thinks and then says angrily: “*You don’t have to be so aggressive with me, there are other ways of saying it*.” This results in an exchange of words between the two women. The nurse replies that she is not being aggressive. The daughter gets angrier and says: “*Have you eaten something funny or is there just something in the air?*” She walks off angrily. [Field note, Norah]

These conflicts also led to healthcare professionals’ perception of some families as aggressive and easily affronted.

### Emotional involvement

Patients’ visitors were strongly emotionally affected by their beloved being in a critically ill state. As a result, they were emotionally involved in patients’ care, which was reflected through a range of ethno-culturally based expressive behaviour, for instance crying hard and falling onto the ground. However, healthcare professionals showed a limited level of compassionate involvement because they wanted to stay purposefully engaged in the care of the patient.

“*The only thing I believe we always need to do is to look after the interests of our patient. And to try and try and put aside the, the emotion in the air as much as you can and focus on what you are trying to do*.” [Interview with nurse Frans]

“*We are not like the nurses in a hospital drama on TV. If we showed that much compassion and had to take everything to heart that much, we wouldn’t last long*.” [Informal conversation between researcher and nurses]

Healthcare professionals’ low emotional involvement was reflected in their distant professional behaviour.

Encounters between visitors’ high emotional involvement and healthcare professionals’ low emotional involvement contributed to conflict. Healthcare professionals found it arduous to have to deal with expressive forms of behaviour and became stressed. This was mirrored by their reserved communication and attitude towards visitors expressing their emotional involvement. It also resulted in the perception of patients’ visitors as irrational or insincere.

A large Portuguese family was occupying the open waiting room for visitors in the corridor during the afternoon visiting hour. They all looked very sad, and some were crying. One woman, the patient’s sister, was crying loudly on a family member’s shoulder. She went for a short walk in the corridor and fell. Dr Vervaecken came and put the woman in a hospital bed and asked her basic questions like “*Are you all right?*”*.* Afterwards I spoke to the doctor in question about this situation, and she said that people who act hysterically ‘get right on her nerves’. “*I can understand that people find it hard and cry, but people who start acting hysterical and* ‘*faking*’*, I have no respect for that, it makes me angry! After all, we also have to keep on going in these difficult situations*,” she said. [Field note, Bento]

Similarly, certain visitors considered healthcare professionals unfriendly as a result of their professional type of involvement.

### Information exchange

Families’ information-seeking strategies, influenced by family structure and ethno-cultural background, hindered healthcare professionals’ professional duty to control the exchange of medical information, which contributed to conflict.

#### Claiming the right to medical information

Family members actively sought information about patients’ situations. Migrant families’ need for information was intensified by the families’ structure. Families were large, and their members were geographically dispersed. Some family members lived in the country of origin, and others lived in the receiving country. Consequently, besides visits from family living in Belgium, patients also received visits from family who lived in the country of origin. Medical information requests from many visiting family members led to feelings of insecurity among healthcare professionals, as they were only allowed to exchange medical information with the patient’s spokesperson (children/legal partner) according to the ICU protocol based on the law.

The patient has a total of three brothers and five sisters. Some members of the family, including one brother and one sister, have come from Morocco to visit the patient. The sister who has come from Morocco visited the patient during the evening visiting hour and tells me she really wants to know how her brother is doing medically so she can decide when to book a ticket back to Morocco. She asks me where the doctor is. I say: “*He isn’t here this evening. You have to ask a nurse.*” The woman returns to the patient and takes his hands in hers. She has tears in her eyes. She speaks to a nurse and asks how the patient is doing. The young nurse simply replies: “*All the patients here are critical*.” The woman presses him a little, saying she really needs information because of booking her return journey to Morocco. The nurse suddenly bursts out with something about the patient’s medical condition to relieve the woman of some of her fears. A nurse who is eating is watching the pair closely and says to the nurse: “*I think you’ve already said enough! You’d better stop!*” Then she spoke to the woman from her seat, with her mouth full: “*Madam, you’re not in Morocco now!*” [Field note, Abdallah]

**Nurse:** “*They* (*the family members*) *didn’t know the whole situation of course, because obviously they didn’t really have the right to that information. And yes*, *it had been said a few times that they had no right to information. So, erm, yes, then they must have got a bit suspicious, maybe for exactly that reason, and erm, they thought things were going on which perhaps weren’t supposed to be.*”

**Researcher**: “*And why didn’t they have a right to it?*”

**Nurse**: “*Well, because that, erm, has to do with the patient’s privacy. If the patient wants to tell them, that’s fine, but for us, medically and legally speaking, they had no right to that information.*” [Interview with nurse Frans]

Moreover, healthcare professionals were unwilling to create confusion within the large families concerning the patient’s situation.

**Researcher:** “*And what do you think the other family members thought about the treatment of, of the patient?*”

**Nurse**: “(…) *But they wanted to know everything in minute detail. Why this? Why that? And they all wanted to hear it individually as well. But that’s impossible. The doctors can’t speak to twenty people individually. Oh, it’s so difficult when you’re explaining something to, to, “How do they see me?” eh, one person says it like this, and the other says it like that. And then if you have to say it to ten different people, and they start discussing it with each other afterwards, it gets even more difficult, I think. Yes.*” [Interview with nurse Conny]

This augmented feelings of suspicion towards healthcare professionals among some family members who felt an ethno-culturally based entitlement to medical information. Moreover the exclusion of family members other than the spokesperson from medical communication sometimes evoked overt intra-familial conflicts in the ICU. These disputes led to frustration among healthcare professionals since they considered them a threat to the care environment. Moreover, according to healthcare professionals, these conflicts interfered with their focus on care tasks.

#### Asking questions

Visitors’ need for information was augmented when patients were no longer able to communicate. This resulted in many relatives asking healthcare professionals numerous questions about the patients’ condition. According to the deontology-based agreements within the critical care team, only one physician is allowed to give medical information to a patient’s spokesperson. Consequently, some nurses gave no medical information to relatives; others gave only little or general medical information. Nurses were afraid to contradict previous information given and did not want to create confusion or false hopes.

**Researcher:** “*Do you, as nurses, also provide information about medical circumstances*?”

**Nurse:** “*No, no, but I haven’t done that for a long time, because it’s not my job to do that and because it sometimes turns out that if you give medical information, you sometimes complicate things for both the family and the doctor, as a nurse. Everyone should stick to their own tasks. So I have learned to say, ‘Sorry, that is not my job. Please ask the doctor’*.”

**Researcher:** “*And in what sense does it complicate things, then*?”

**Nurse:** “*Erm, well, because, erm, how someone, how a certain situation is explained, someone says, the nurse, and then the doctor doesn’t know what has already been said, or how it has been explained. Different people explain things differently. It is already hard for people to understand in a situation like this and then they hear different opinions, different perspectives. And erm, in the long run they just get completely confused, as it were (…) Everyone has their own way of seeing things as well, or explains things in their own way, and in very, in critical situations like intensive care it is best, isn’t it, for one person, for everyone to see things more or less the same way. And I know from experience that the fewer the better, hmmm, the fewer people involved in giving explanations, the better. One, one doctor and, well, there you are, that’s best*.” [Interview with nurse Veerle]

Furthermore, many questions from family members drew healthcare professionals’ focus away from their own job responsibilities and codes of conduct, which could result in a minimum of information exchange. Some family members considered healthcare professionals unfriendly when they asked them for medical information.

**Patient’s son**: “*Is it normal for her to be so disoriented?*”

**Nurse**: “*Visiting hour is over, sir… I have to ask you to leave now*.”

**Son**: “*Yes, I know, but will the doctor be here tomorrow?*”

**Nurse**: “*Visiting hour is over… Visiting hour is 6 p.m. to 7 p.m.*”

**Son**: “*Yes I know when visiting hour is… She has been here for a long time… I know how the hospital works… Will the doctor come tomorrow?*”

**Nurse**: “*Sorry, visiting hour is over… You have to leave now.*”

The son gives up and walks away from the nurse. He whispers the following to the researcher: “*She’s not very nice.*”

**Researcher**: “*Why?*”

**Son**: “*The way she talks… I know perfectly well when visiting hour is.*” [Field note, Fadila]

#### Seeking a second opinion

In cases where some patients’ physicians started doubting the efficacy of the whole treatment at a certain point, family members sought a second opinion. Families considered it their responsibility to figure out whether the right medical care was offered to the patient and, if not, to seek alternative treatment options. The search for a second opinion, reinforced by group pressure among family members, was perceived by some doctors as a threat as they do not like to share detailed medical information with third parties for reasons of professional secrecy. Moreover, seeking a second opinion can be considered a threat to doctors as their medical expertise and rationality was implicitly questioned by this act. The feeling of being hindered when seeking for a second opinion led to stress among family members. As a result, the process of seeking a second opinion stimulated mutual distrust between families and doctors, which in turn contributed to conflict.

Patient’s son:

“*And I would like a second opinion on my father’s treatment* (*…*)*. I asked the doctor on duty if I could ask him a few questions about my father’s medical condition, and he gave a very offensive answer. After the third question, he said he no longer wished to answer, and that he would only discuss this information on the telephone with the neurologists, ‘with another doctor’ as it were.* (*…*) *I need specific information to pass on to a neurologist friend. I have already asked the doctors to give me the results of the medical tests so that I can pass them on to the neurologist that the family know, but they don’t want to.*” I ask him why the doctors don’t want him to have the results of the medical tests. He says: “*They don’t want the information to be leaked to third parties. But I’m not a third party, I’m the son, I have a right to it. I am not going to give the information to just anyone… But they want this information to be passed from doctor to doctor, they insist on only communicating* ‘*with other doctors*’*.*” The son says all this calmly, but I see that he is annoyed. I ask why he wants to do this. He says: “*Then we will know what steps we can take if they can’t help him here.*” (…) The son apparently feels that he is to a great degree responsible for his father’s care. [Informal conversation between patient’s son and researcher, Field note, Quintus]

**Doctor**: “*I have the feeling the family don’t trust us very much… They want a sort of second opinion from that neurologist friend of theirs… I don’t want to give the son the medical file because the doctor’s notes we make here are only the doctor’s ‘opinion’, I’d prefer to discuss it with another doctor because we don’t want the information being leaked to third parties.*” [Conversation between Dr. Vervoort and researcher, Field note, Quintus]

### End-of-life decision making

Physicians’ view of end-of-life decisions as medical decisions became endangered by families’ desires to be actively involved in end-of-life decision making, influenced by ethno-culturally based values, reinforced by group pressure within families and migration-related factors.

#### Families’ claim for involvement in end-of-life decision making

Families considered a decision to withdraw therapy an act of ‘killing’ the patient and claimed power over decision making. Several migration-related factors served as additional sources of hope for families. First, family members’ history of migration fostered the development of infinite expectations for cure. The presence of advanced technological equipment and highly specialized staff in Western hospitals led to the idea of the existence of an unlimited field of medical possibilities, which was felt to be absent in the country of origin.

**Patient’s husband:** “*But at the weekend they said she was okay. And now you say she’s not*.” [man begins to cry]

**Doctor***:* “*We have to wait for the situation to stabilize. Her situation has always been critical. That is what I have said in all our conversations. We are doing all we can to help her*.”

The man keeps on saying that his wife was doing okay at the weekend. The doctor repeats that she has always warned him that her situation is very bad, and that it might get worse. The discussion continues and the doctor starts to stress out and becomes upset. The doctor starts to talk about the possibility that the patient may be near death.

**Doctor**: “*We have done what we can. She might die.*”

**Husband**: “*She might die? This is the biggest hospital in Belgium. She ‘has’ to get better. I won’t let the situation get any worse*.”

**Doctor:** “*It is possible.*” [upset]

The man starts crying more and wants to continue the discussion. [Field note, Amrani]

Second, some family members used their ethnic origin or religion as source of infinite cure expectations. God/Allah was seen as having the capacity to decide over the life and death of the patient, or only members of families’ particular ethnic group were seen as able to sense the impending death of the patient, not doctors.

She (the patient’s godchild) repeats that God decides when someone will die. Then she says that ‘Moroccans’ see in someone’s eyes when they are going to die. “*Moroccans see death in patients’ eyes, they can see how long a patient has left to live.*” I ask her how they can see this. She cannot come up with a clear explanation of this. [Conversation between researcher and family member of patient, Fadila]

Furthermore, the considerable size of most families led to group pressure among its members to assure the continuation of life-sustaining therapy. On the other hand, healthcare professionals considered withdrawing futile medical therapy a medical decision, because they considered that they had the necessary medical expertise and an objective and rational point of view. Moreover, healthcare professionals tried to follow the deontological code which prohibited futile medical therapy. Consequently, when doctors started talking to patient’s spokesperson about a decision to withdraw therapy, trust between the two parties decreased.

**Doctor: “***He needs to react to the treatment on Monday.*”

**Man (son of the patient):** “*And what if there is no positive reaction?*”

**Doctor:** “*We will not continue to treat him this way*.”

**Man:** “*And what will you do then?*”

**Doctor**: “*We will take him off the machines.*”

**Man***:* “*So if there is no improvement you will turn off the machines… So you’re saying that there are no treatments left and that you intend to stop? … I’m not speaking for myself, but I already know that that isn’t what the family will want.*”

**Doctor**: “*It is not up to you to decide. This is a medical decision.*”

**Man**: “*What about the family then? The only thing you do is talk to the family?*”

Doctor gives no answer to these two questions (…)

**Man***:* “*I have called my family, and we are going to talk next week.* (*…*) *With what has been said, we don’t know.* (*…*) *Maybe there are other options… There is nothing more to be done, okay… Maybe we can take him with us or something else… Then it will be up to ‘us’ to decide… I just want to know what you plan to do.* (*…*) *I am not just speaking on my behalf, you see… There’s the whole family… It isn’t up to me to decide…* (…) *‘Stopping the machines’ means we would ‘kill’ him*.”

**Doctor:** “*It doesn’t mean ‘kill’… Stopping the machines is not euthanasia: we are not going to give him any medication that will lead to his death, we are letting his body decide for itself… That is a medical decision… If someone has been in intensive care for three weeks and the treatment isn’t helping, we have the ‘right’ to stop the machines.*” [Conversation between doctor Robberechts and son of patient, Field note, Quintus]

## Discussion

This ethnographic study investigates factors contributing to conflicts between healthcare professionals and family members from ethnic minority groups in an intensive care setting in Belgium. During our study, several conflict situations occurred with regard to visits, bedside care activities and care requests from family members, their emotional involvement, their requests for information, and active involvement in end-of-life decision making. Analysis of our deviant case also suggests that such conflicts are more likely if patients frequently receive many visitors. When examining the situation in great depth, we found that these conflicts were basically related to differences in the participants’ views on what constitutes ‘good care’ based on different care approaches, and the - often incompatible - associated expectations. However, our study also suggests that structural, functional characteristics of the ICU approach substantially contributed to the emergence of conflicts out of these differences, and probably played a more important role in the conflict situations than participants’ ethno-cultural differences as such (see Additional file 1).

Conflict in critical medical situations in a multi-ethnic context is an under-researched topic. The few studies that deal with this subject directly approach it through interviews with one of the parties (see for example [16]). Other studies only touch on this subject indirectly in the context of researching the experiences or needs of parties involved in intercultural communication [15, 18, 19, 22, 35, 36]. As far as we know, this is the first study where conflicts in a multi-ethnic context in the ICU were investigated by means of an ethnographic research design. A study design of this kind is one of the most appropriate ways to gain nuanced and deep insight into complex situations such as conflicts in a multi-ethnic critical care context. Ethnographic, day-by-day observations on the ward allow the study of conflicts as they actually occur and from the perspective of the different people involved, without the risk of reducing the problem to ethnic stereotyping. A weakness of this research could be the fact that the fieldwork was done in only one ICU. Therefore transferability to other situations must be done with caution. However, by spending a lot of time in a variety of situations (with ten patients and their families, and all the healthcare professionals) it was possible to build sufficient trust and to gain rich knowledge about this sensitive topic. There is no specific reason to believe our findings are not valuable for other comparable contexts.

A central finding of our study is that healthcare professionals as well as the families included all wanted the patient to receive the best care possible. However, what these actors define as ‘good care’ is based on their care approaches, which share common elements but also differ substantially in certain areas. We found that families’ views on good care were mainly inspired by a holistic lifeworld-oriented approach, whereas healthcare professionals’ views on good care seemed to be based predominantly on a biomedical care model. Health, from the families’ perspective, means the general wellbeing of their loved ones in several areas. Family members were not only concerned with patients’ biological needs but at least as much with psychological, social and even religious needs, and not only of the patient but of their whole family. Good care from this point of view can take very diverse forms, implying a set of specific social role expectations, including visiting the patient, giving bedside care to the patient, seeking exhaustive medical information, participating in end-of-life decision making, and remaining hopeful. The biomedical paradigm, on the other hand, considers health primarily as the absence of bodily decline, resulting from a somatic pathology. According to this model, healthcare professionals primarily focus on the diagnosis, treatment and cure of somatic problems, caused by biological processes and expressed in signs and symptoms [37]. Giving good care, from this point of view, also entails certain role expectations, e.g. making an accurate diagnosis, removing the disease as well and quickly as possible with great scientific competence, maintaining professional distance, attaching great importance to regulations, communicating with relatives in a controlled, limited, functional and structured manner, and believing in the central responsibility of physicians in the medical decisions to be taken (e.g. end-of-life decision making).

Although, in general, a change towards a more holistic approach to healthcare can be observed over recent decades, this is still less obvious in the daily practice of medicine, especially high-tech contexts such as that of the ICU [7, 37]. The care context of the ICU is characterized by the performance of lifesaving care tasks, making life-and-death decisions, a technological orientation, a specific regulatory framework (Belgian law on the rights of the patient, deontology, agreements within the team), time pressure, uncertainty, ambiguity, and anxiety [38, 39]. These characteristics strengthen healthcare professionals’ biomedically inspired views on what constitutes good care, alienating them further from families’ views on good care. Families’ holistic views on offering good care, on the other hand, seemed to be strengthened by the specific characteristics of the ethno-familial care context of the observed minority groups. First, the structure of these families, i.e. large and transnational social networks, evoked group pressure among its members to offer care practices, seek medical information, and demand involvement in end-of-life decision making. Moreover, families’ structure impeded clear communication within the network around the patient, which led to confusion about patients’ medical situations, affecting families’ need to seek information. Second, families’ shared ethno-cultural background, involving socio-cultural norms, values, forms of behaviour, communication and practices, intensifies the significance of ethno-culturally based care practices, expressions of involvement, information-seeking strategies, and the need to participate in medical decision making. Third, experiences of discrimination and integration-related role expectations affected the importance of families’ ethno-cultural forms of care. Studies have shown that experiences of discrimination and expressions of ethnic identity are positively correlated [40]. Furthermore, family members lacking knowledge of the language and/or culture of the receiving society have learned to be dependent on other family members who act as their translators or assistants during daily life. As family members have internalized these roles, they will also take on this function during the performance of care activities in critical medical situations. Fourth, migration-related factors, i.e. families’ history of migration, their different ethnic origin and religion, functioned as additional sources of hope and influenced families in claiming end-of-life decision-making power. Some studies have pointed towards the role of racism and ethnic prejudice in tensions in a multi-ethnic ICU (see for example [16, 41]). However, in our study, these factors did not come to the fore as main contributors to staff-family conflict.

Previous research has shown that healthcare professionals (i.e. physicians) generally identify a situation less often as conflict-laden than relatives [6], suggesting the presence of hidden or unrecognized conflicts during critical care [42]. However, our findings show that in a multi-ethnic critical care setting conflicts were very overtly present because they were explicitly recognized by both relatives and healthcare professionals, and many were very visible and audible on the ward. Furthermore, where relative-staff conflicts in general tend to be centred around moments of crisis (end-of-life decision making, patients’ deaths) [6, 7], we found that in a multi-ethnic critical care context, conflicts tend to be present during multiple care phases, affect a broad spectrum of care aspects and may assault the core of actors’ identities. This might further complicate end-of-life decision making in a multi-ethnic critical care context. We believe that conflicts in the end-of-life decision-making phase can be diminished if conflicts in the phases prior to end-of-life decision making can be reduced.

## Conclusions

Although families’ different ethno-cultural backgrounds play an important role in conflicts between healthcare professionals and migrant family members in the ICU [16], our study clearly shows that looking ‘exclusively’ at the contribution of actors’ different ethno-cultural background to explain conflict obscures the context-specific features of these conflicts. How ethno-cultural differences are bridged during communication between relatives and healthcare professionals is largely dependent on the ICU’s own approach (structure/policies/work habits). During conflict, ethno-cultural differences were not adequately dealt with by the ICU’s approach. As a result, ethnic difference seems to contribute to conflicts only in confrontation with a triggering contextual structure, for example with regard to professional responsibilities and work context. Consequently, the role of ethno-cultural differences must not be overstated in staff-family conflict in a multi-ethnic critical care context.

Effective conflict prevention should not only focus on ethno-cultural difference but should also take the structural, organizational characteristics of the critical care context sufficiently into account. ICU policy should stimulate the coexistence of biomedical and more holistic views on health and care among staff. Consequently, we argue that giving healthcare professionals training in cultural competency, i.e. programmes aimed at increasing healthcare professionals’ intercultural awareness, knowledge and communication skills [43], is insufficient on its own to avoid conflict. Developing and implementing organizational measures which allow maximal collaborative communication both within the ICU team and between relatives and staff in every care phase is crucial to prevent conflicts between staff and relatives in a multi-ethnic critical care context. ICU policy should give healthcare professionals the capacity to learn to communicate with relatives effectively on the work floor, should stimulate sufficient information provision to relatives and allow a certain degree of family participation in patients’ care. This can be done by questioning ward habits, unwritten rules and formal policies (e.g. visiting policies, healthcare professionals’ work hours). Allowing the creation of a partnership between staff and relatives on a structural level can reduce the potential dividing influence of ethno-cultural differences during critical care. Furthermore, a better integration of palliative care and ethical consultants is advised in the end-of-life decision-making phase. Further research is greatly needed to develop and test specific conflict prevention measures in a multi-ethnic critical care setting.

## Key messages

- Conflicts were primarily related to healthcare professionals’ and families’ different views on what constitutes good care.
- Conflicts are not exclusively related to actors’ ethno-cultural differences as these differences contributed to conflicts only where a triggering critical care structure was present, e.g. healthcare professionals’ job responsibilities and work context.
- Effective conflict prevention should not only focus on ethno-cultural difference but should also take the structural, organizational characteristics of the critical care context sufficiently into account.

## Appendix

### Topic list for formal in-depth interviews

- Pathology
- Experiences with communication (patient - relatives - intra-team)
  - ○ Difficult communication/disagreements (if applicable)
- Decision making
  - ○ Role - nurse - doctor - patient - relatives
  - ○ Course
  - ○ Difficult communication/disagreements (if applicable)
  - ○ Final medical decision
- Care for patient
  - ○ Positive and negative experiences
- Death of patient
  - ○ Communication with relatives
- Prevention of difficult communication with relatives

## Additional file

Additional file 1: Figure S1. Conceptual model on conflict between healthcare professionals and families from ethnic minority groups in the ICU. (PDF 157 kb)

## Abbreviations

ICU: intensive care unit
