# Supplementary material for: Conflicts between healthcare professionals and families of a multi-ethnic patient population during critical care: an ethnographic study
Source: Crit Care. 2015 Dec 22;19:441. doi: 10.1186/s13054-015-1158-4 (PMC4699338; doi:10.1186/s13054-015-1158-4)

Figure: Conceptual model on conflict between healthcare professionals and families from ethnic-minority groups in the ICU

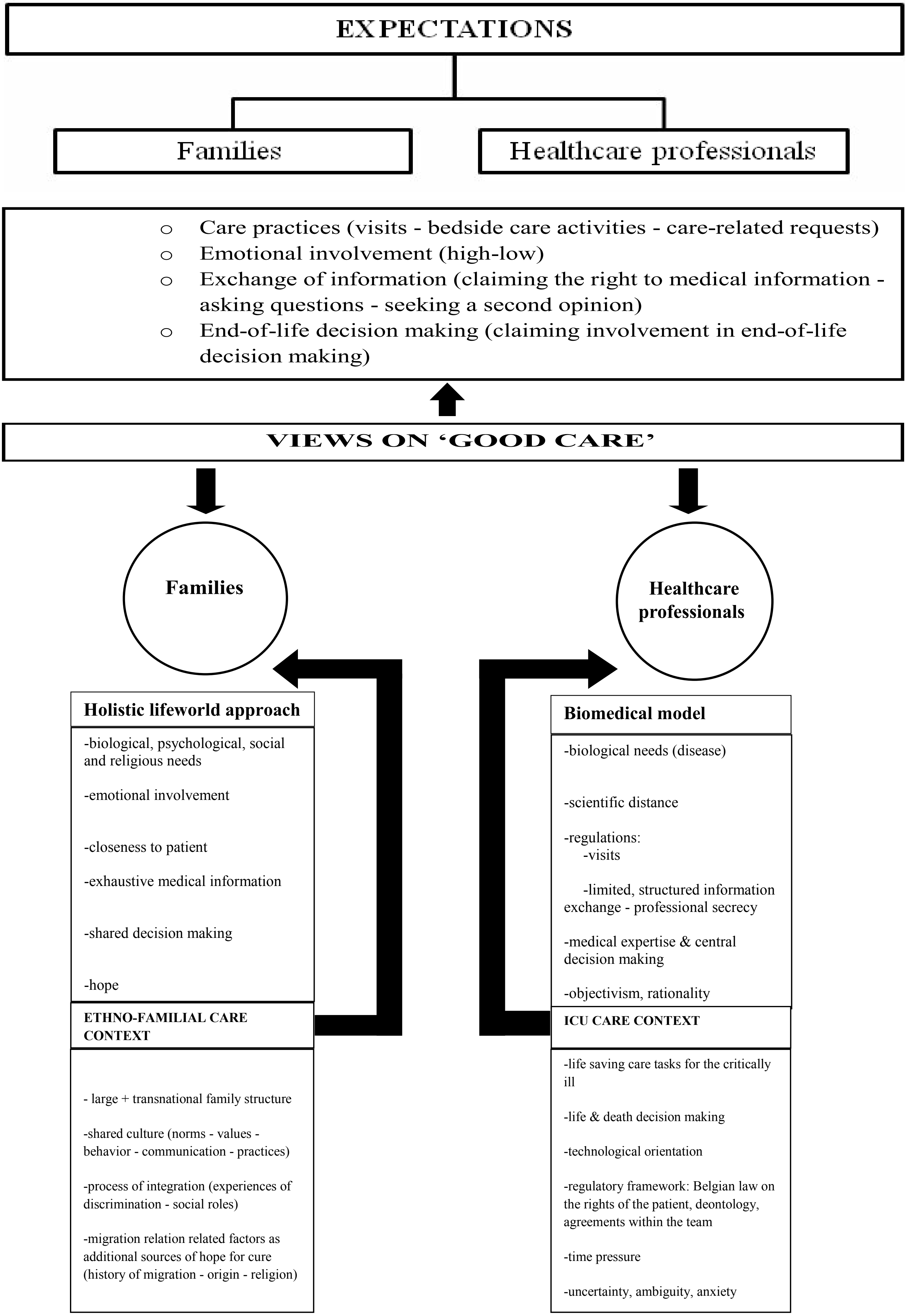

Supplement: Additional file 1: Figure S1. — Conceptual model on conflict between healthcare professionals and families from ethnic minority groups in the ICU. (PDF 157 kb) [file 13054_2015_1158_MOESM1_ESM.pdf]
